# Inoculation with Tartarized Antimony

**Published:** 1846-01

**Authors:** 


*Inoculation with Tartarized Antimony.*—It is well known that tartarized antimony constitutes one of the most active agents of revulsive medication; all practitioners have employed either Autenreith's pommade or stibiated plasters, and all have observed the development of specific pustules consequent on these applications. These pustules are developed irregularly; sometimes the eruption is too slight, and at others it is confluent, and then causes the formation of wounds, which are difficult to cure. To avoid these inconveniences, M. de Bourge de Rollot has had recourse to stibiated inoculation. The following is the description of his new plan (*Répertoire du Progrés Médical, par MM. Wahu et Quénot*):—

"A small quantity of porphyrized tartarized antimony is placed on a glass plate, and mixed with a little water or oil, just in the same manner as is practised with the dry vaccine lymph when it is about to be used. A lancet is charged with this mixture, which should be thick enough not to flow on its point, and the requisite number of punctures is made in the part selected, as far apart from each other as may be deemed necessary, according to the indications to be fulfilled; in ordinary cases, I generally leave a space of from three to four centimetres between each. These punctures soon inflame, and the slightly pustulous inflammation which ensues, and which would soon disappear if let alone, is, on the contrary, soon changed into pustules, which acquire a more or less considerable size in proportion as their surface has been more or less frequently and carefully, and for a longer or shorter time, covered with a small quantity of tartar-emetic mixture, either the aqueous or the oily, which was used for their production. I use for that purpose a small wood spatula, and I repeat the stibiated application on the pustules, evening, morning, and noon, for two, three, four, or five days, &c., according to the intensity of the inflammation which I wish to excite. When the affair is urgent, I would advise applying a fresh layer of the tartar-emetic compound on the pustules every two hours, by which means they will be developed so much more quickly.—*Med. Times*, from *Bouchardat's Annuaire* for 1845, in *Med. News*.

